# Digestibility of Boiled Milk

**Published:** 1890-02

**Authors:** 


### Digestibility of Boiled Milk.

It is now very generally recognized, both by medical men and by the more highly educated section of the community, that it is a wise precaution to boil both water and milk before using them as beverages, and the practice is becoming very common. The growth of pathogenic organisms in these fluids, epsecially milk, is often very rapid, and thus disease may be transmitted from one place to another. The temperature of boiling water puts an end to the life of the microbes, and also to the danger of infection. Another reason why boiled milk is so much used, especially in infant feeding, is that it is supposed to be more easily digestible than fresh milk. If, however, we can draw correct deductions from dogs to babies, it would now appear that this belief in the superior digestibility of boiled milk is founded on error. Dr. Randnitz, of Prague, has recently published, Hoppe-Seyler's *Zeitschrift fur Physiologische Chemie*, certain very striking experiments on this subject. He admits what any one may confirm for himself, that milk that has been boiled does not, on cooling and the subsequent addition of rennet, form a large coherent clot as does fresh milk; but a flocculent precipitate of casein is produced instead. He shows, however, by analysis of the milk itself, and of the urine and fæces, that much less nitrogenous material is absorbed from milk that has been boiled than from the same milk when fresh. The digestibility of fat is apparently unaltered by boiling. The following figures, however, illustrate the fact just alluded to as to the difference of digestibility of the proteid materials: In three days 15.6 grammes of nitrogen were given in the form of fresh milk; of this quantity, 13.3 per cent. was found in the fæces; the nitrogen of the urine accounted for 77.3 per cent., so that 9.4 per cent. was retained in store by the growing animal. The animal was next fed on boiled milk; and 10.4 grammes of nitrogen was given in that form in two days; 18.6 per cent. of this was found in the fæces, 75.7 in the urine; so that only 5.7 per cent. was assimilated. The belief in the superior digestibility of boiled milk is, however, so widespread, we should like to hear of the confirmation of the above remarkable results before we recommend mothers to leave off what is, from other points of view, the very praiseworthy custom of boiling the milk they give to their children.—*British Med. Journal.*

An Essentially Woman's Hospital.—A hospital devoted to diseases of women and under the patronage of the Princess of Wales is to be established in England. The special feature of the institution is that all services to the patients, both professional and otherwise, are to be rendered by women.

